# Operational flexibility impact on hospital performance through the roles of employee engagement and management capability

**DOI:** 10.1186/s12913-023-09029-y

**Published:** 2023-01-09

**Authors:** Main Naser Alolayyan, Mohammad Sharif Alyahya

**Affiliations:** Department of Health Management and Policy, Faculty of Medicine, Jordan University of Science and Technology, Irbid, Jordan

**Keywords:** Hospital performance, Operational flexibility, Employee engagement, Management capability, Hospitals

## Abstract

**Background:**

Very limited empirical research has been done on operational flexibility management in the healthcare industry, especially in hospital settings. This study aimed to propose a model of the effects of operational flexibility on hospital performance through management capability and employee engagement as mediating variables.

**Methods:**

The proposed model is validated through an empirical study among 480 clinical and administrative staff from five hospitals in Jordan. Structural equation modeling and confirmatory factor analysis were the main techniques used to validate the model and examine the hypotheses.

**Results:**

Operational flexibility was demonstrated to have a positively significant impact on hospital performance, management capability, and employee engagement. Employee engagement was demonstrated to positively impact hospital performance. Management capability had a significant result on hospital performance without having a clear impact. In addition, management capability and employee engagement played a major role as partial mediating effects between operational flexibility and hospital performance, and there is a role for employee engagement as a partial mediating effect between management capability and hospital performance.

**Conclusion:**

Significant progress has been achieved in hospital management, especially in terms of operational flexibility, management capability, and staff engagement.

**Supplementary Information:**

The online version contains supplementary material available at 10.1186/s12913-023-09029-y.

## Introduction

Operational flexibility means the ability of firms to deal with external and internal challenges, thereby creating competitive opportunities and the ability to reduce losses [1, 2]. Technically, operational flexibility refers to three dimensions - input flexibility, processing flexibility, and output flexibility - for the service delivery system [3]. Dimensions of input flexibility and output flexibility allow the firm to communicate with distinguished suppliers and all types of clients, thus obtaining a value chain that is positively reflected in the organization’s productivity [4].

The main focus of this research paper is on the hospital services sector. Nowadays, hospitals face many changes in the internal and external environment, such as epidemics, changes in health policies, and revolutionary changes in health technology. These changes create a situation of difficulty in predicting future needs and demands in the health sector, which affects the level of services in the hospitals. Operational flexibility gives a unique opportunity to face these changes and uncertainties with high efficiency, which affect the performance of the hospital in general. Hospital flexibility depends on a flexible workforce, resources’ flexibility, working hours’ flexibility, and suppliers’ flexibility. Operational flexibility can enhance the hospital’s capability to respond to patients’ preferences, employees’ needs, and changes in healthcare needs by offering the required supplies (such as beds, drugs, and consumables) and suitable infrastructure [5, 6].

However, the most important challenge remaining is to achieve a high level of operational flexibility to deal with multiple changes and a high level of patient expectations. The hospitals need important management tools that support the idea of operational flexibility to face internal and external changes, which is reflected in the hospital’s performance. So, this study will focus on the mediating role of management capability and employee engagement as important management dimensions to support the relationship between operational flexibility and hospital performance.

Operational flexibility, as one of the basic types of resilience, has become essential to respond quickly and effectively to dynamic environments, thereby improving its performance. Flexibility means the ability to quickly adapt with minimal penalties in terms of time, cost, effort, or performance as the situation changes [7].

Operational flexibility refers to the ability to respond proactively or reactively to uncertainties in the business environment. This ability has a number of dimensions that may vary in importance. There is growing concern about the idea of flexibility and its applicability to operations management. Flexibility has long been recognized as an important factor in a competitive industry characterized by high competition, short product life stages, rapid changes in customer preferences, increasing technical innovation, etc. Generally speaking, resilience refers to a firm’s ability to deal with or adapt to environmental uncertainty, thereby creating opportunities for obtaining a long-term competitive advantage [8].

In cases of epidemics such as the COVID-19 epidemic, operational flexibility played a major role in adapting to the new situation created by the epidemic. Most healthcare systems or healthcare service organizations that implement the philosophy of operational flexibility were able to adapt to the reality of the new epidemic and achieve remarkable results compared to the other healthcare systems. On the other hand, a healthcare service system that is ignorant or lacks the skills to enhance the operational flexibility of healthcare organizations meets many challenges and fails to deal with epidemic situations and their impacts. The COVID-19 pandemic was a real and important experience that showed the importance of operationally flexible processes to face internal changes and external challenges for healthcare institutions [9].

### Theoretical framework and hypothesis development

Operational flexibility refers to the organization’s ability to deal with its business environment, either proactively or reactively; this ability has a positive impact on the organization’s performance [10]. Organizations that have high levels of flexibility have the ability to produce unique and new products very quickly, adapting to the global technological acceleration, which also enables them to enhance the amount of production without affecting the cost, time, or performance [11]. Implementing such flexibility requires resources prepared for this task (flexibility), including human resources with the highest degree of coordination and the lowest risks and costs [12, 13]. According to Alolayyan, Ali [14], the sustainability and development of the service depend primarily on the level of flexibility of the various operating systems to respond to changes both externally and internally. It also enhances the development of alternative solutions and scenarios of potential changes. As a result, we can conclude that operational flexibility has a positive impact on the performance of organizations.

**H1: There is a positive direct impact of operational flexibility on hospital performance.**

When the philosophy of flexibility is implemented in the work environment, employees will have sufficient flexibility in their work, which achieves a high level of responsibility and work-life balance [15]. This in turn enables employees to plan their own ways to improve their capabilities, time, energy, and attention to their work effectively, which increases independence, work control, and efficiency, and allows them to be more involved and engaged in the work [16].

Psychological relationships, competence, and independence, as necessary psychological needs, may lead to employees’ integration and development. It has been argued that the psychological needs of employees build a sense of self-esteem and awareness of their strengths, which results in more interest, authenticity, participation, and engagement [17–19].

The relationship between intrinsic motivation and flow experiences supports the importance of engagement as a psychological need for independence. Flow experiences are the holistic and overall positive feelings that employees feel when they participate extensively in their job tasks [20]. Organization leaders can benefit by engaging junior employees to secure a long-term mutual psychological contract and creating an appropriate environment for commitment, creativity, and process-oriented organization [21].

**H2: There is a positive direct impact of operational flexibility on employee engagement.**

Operational flexibility has a positive impact on developing operational design and quality improvement and ensuring proper handling of procedures by a capable management [3, 22]. Management capability is the ability to apply high managerial skills, competencies, and knowledge to create an interactive environment to integrate the various resources of the organization, tangible and intangible, to maximize the benefit from employees’ engagement with different operating processes [23–25]. Better managerial capability leads to robust and improved organizational performance. Based on this background, we suggest that optimal organizational performance can be effectively achieved through better operational flexibility, managerial capability, and employee engagement.

**H3: There is a positive direct impact of operational flexibility on management capability.**

Work engagement is a satisfactory state with high mental integration that has a positive link to the work; it positively impacts employee performance through a high understanding of work requirements, activity, and efficiency. It has been found that employees’ engagement is an important success factor for enhancing the organization’s performance [26, 27].

**H4: There is a positive direct impact of employee engagement on hospital performance.**

Healthcare settings managed by highly qualified clinical managers with high management capabilities have better management practices [24]. Mascia and Piconi [28] studied the relationship between the functional methods of managers and their performance in Italian hospitals and found that CEOs with significant experience in a large number of health care systems and hospitals with discrete systems achieve high levels of managerial performance.

**H5: There is a positive direct impact of management capability on hospital performance.**

It has been found that the principles of management, management capabilities, and the management process affect employee engagement positively and directly. Organizations need to apply a distinctive management approach, represented by the ability of managers to employ their distinctive capabilities to design various organizational processes and an effective strategy that leads organizations to higher levels of innovation and to obtain more effectiveness, productivity, and growth [24, 25, 29].

**H6: There is a positive direct impact of management capability on employee engagement.**

#### The mediating role of employee engagement and management capability

There are significant limitations to research studies on employee engagement and management capability as a mediating factor between operational flexibility and hospital performance. The impact of the mediating factor is based on the importance of the mediator as a supporter of operational flexibility applications, which ultimately contributes to improving the productivity of individual and organizational hospitals. Same thing for the mediating role of employee engagement between management capability and hospital performance.

The limited studies on this subject give great importance to this study from the literary and productive sides of the medical sector. To this end, we propose the following:

**H7: There is an indirect impact of operational flexibility on hospital performance through employee engagement.**

**H8: There is an indirect impact of operational flexibility on hospital performance through management capability.**

**H9: There is an indirect impact of management capability on hospital performance through employee engagement.**

**Fig. 1:**
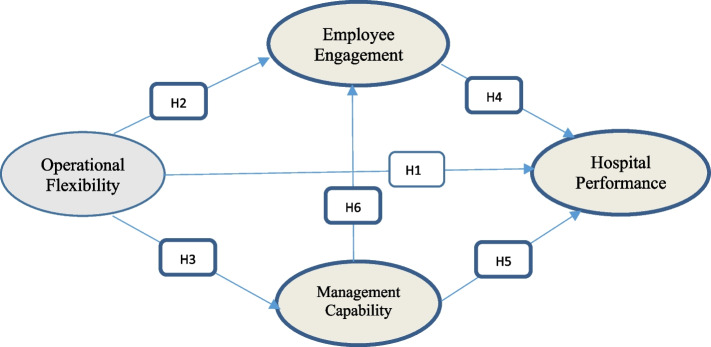
Research model

Please see Fig. 1 to explain the entire research hypothesis.

## Method

### Description of source population

The study was conducted in five public hospitals in Jordan. The research participants were medical managers and leaders, supervisors, senior administrative staff, the head of the medical unit, specialists and resident doctors, and nurse supervisors.

Regarding the inclusion and exclusion criteria, all members of the study population have a big role in designing the hospital’s strategies and policies and, at the same time, make daily contributions to the decision-making process, which leads to formulating the mechanisms of operational flexibility in hospitals and its reflection on their performance, management capability, and employee engagement.

The study population was carefully selected to be able to answer the questions of the study instrument through their experiences and daily activities.

### Samples size determination method

For determining the appropriate sample size, the size of the population was identified through the hospital administration departments of the hospitals. However, one of the most important foundations for determining the appropriate sample size is the type and goals of the research being conducted. There is also an important general rule for determining the statistical strength (i.e., sample size), which is “providing five observations for each independent variable” [30]. In order to achieve this goal, a stratified random sample method was used to recruit the study participants.

### Sampling techniques and sampling procedures

To empirically validate the conceptual framework and test the hypotheses, a cross-sectional study design was employed. Stratified random sampling was used. Approximately, the population size was 1500; among them, seven hundred (700) were invited proportionally to participate in the study. The researchers used two methods to collect data: the first method was a semi-structured interview questionnaire, and the second method was a paper copy of the questionnaire given to the study respondent and then collected from the respondent the next day. Six hundred fifty respondents completed the questionnaire, with a respondent rate of 92.85%, but 170 were excluded. This left our sample size at 480 for analysis. One hundred seventy questionnaires were excluded due to a number of issues related to incomplete filling out of the questionnaire, loss of the questionnaire, damage to the questionnaire, lack of clarity in the answers, or failure to return the questionnaire. These issues were dealt with carefully in order to achieve the quality of the data included in the research study.

### Study population and study sample

Table 1 shows that out of the 480 participants, 295 (61.5%) were female and 221 (46%) were between 20 and 35 years old. About 64.8% of the participants had a managerial responsibility, while 22.9% were resident doctors and specialists, respectively.

**Table 1:** Characteristics of participants

Demographic Variables	Frequency (N)	Percent (%)
**Name of the hospital**		
Hospital number 1	132	27.5
Hospital number 2	124	25.8
Hospital number 3	72	15.0
Hospital number 4	111	23.1
Hospital number 5	41	8.5
**Profession**		
Medical managers and leaders	27	5.61
Supervisors	182	37.92
Senior administrative staff	62	12.92
Head of medical unit	40	8.35
Specialist and resident doctor	110	22.9
Nurse supervisors	59	12.3
**Education level**		
Diploma	73	15.2
Graduate degree	170	35.4
Postgraduate	144	30.0
Higher Specialty in Medicine	93	19.4
**Gender**		
Male	185	38.5
Female	295	61.5
**Age**		
20- less than 35 years	221	46.0
35- less than 50 years	181	37.8
50 - less than 65 years	70	14.5
65–80 years	8	1.7
**Experience**		
Less than 2 years	50	10.4
2–5 years	98	20.4
5–10 years	129	26.9
10–25 years	203	42.3

Total number of respondents = 480

### Means of data quality control

- The researchers used five traits to achieve data quality control:
- *Accuracy*: The study sample was selected with great accuracy so that all study respondents are fully able to answer the questions of the study tool through their experience and knowledge, which is reflected in the quality of the study data.
- *Completeness*: The researchers followed a method that relied on excluding any questionnaire that was not ideally complete, and the collected sample was sufficient according to scientific research methodologies.
- *Reliability*: The study instrument components were selected from previous studies so that each part of the questionnaire achieved reliability and validity in these studies. Also, this study verified the reliability and validity by statistical analysis using Cronbach’s alpha, Composite Reliability (CR), and Average Variance Extracted (AVE).
- *Relevance*: In this study, there is an integration between the objectives of the study, the study instrument, and the study community, and this is evident in the sequence of the research methodology used.
- *Timeliness*: The data was collected in January and February of 2020, before Corona, and permission was sought before distributing the questionnaire or conducting the semi-structured interview, giving all respondents sufficient time to complete the study instrument in an ideal manner [31].

### Variables of the study

In this study, there are four variables, which are as follows:

1. Independent variable: operational flexibility; contains three dimensions.
2. Independent variable, hospital performance; contains six dimensions.
3. Two mediating variables: management capability and employee engagement.

### Types of questionnaire

The quantitative method was applied through a cross-sectional study; the researchers used a paper research questionnaire that contains close-ended questions, and the respondents’ answers were based on the Likert scale.

### Survey instrument

The survey instrument was developed based on previous work and updated using data from recent research on operational flexibility, employee engagement, management capability, and hospital performance. The questionnaire is composed of questions relating to four constructs, namely: operational flexibility, employee engagement, management capability, and hospital performance. The conceptual constructs are deemed to be the most comprehensive practices for measuring the impact of operational flexibility on hospital performance through employee engagement and management capability, thus making them suitable for the research objectives of this study. The operational flexibility measures consist of three dimensions with 13 items, while the hospital performance measures construct consist of six dimensions with 27 items. The management capability measure consists of six items, and the employee engagement measure consists of 12 items. Minor modifications were made to some items in the original scale to adjust for semantic meanings. Items scales for (hospital performance) were based on a five-point Likert scale, ranging from “strongly disagree” to “strongly agree”. Items scales for (operational flexibility) were based on a seven-point Likert scale, ranging from “completely disagree” to “completely agree”. Items for employee engagement were based on a five-point Likert scale, ranging from “strongly disagree” to “strongly agree”. Finally, management capability measure based on a seven-point Likert scale rating ranging from (1) strongly disagree to (7) strongly agree was used. Before data collection, the questionnaire items were discussed in depth with three experts in healthcare management, and their comments were considered in the final version.

### Statistical analysis

Several statistical analysis techniques were used in this study. First, descriptive statistical measures such as the mean and standard deviation were calculated. Then Cronbach’s alphas and inter-item correlation were identified to examine internal consistency reliability. The analysis of confirmatory factor analysis was also used to determine the validity of the elements of the study questionnaire. To verify the validity and reliability of the study instruments, both the composite reliability (CR) and average variance extracted (AVE) were used.

### The hypothesized model and the modeling strategy

The model to be tested postulates a priori that operational flexibility has, indeed, influenced hospital performance through employee engagement and management capability. As we believe that this is a rare attempt to quantify operational flexibility for medical service industries, especially in teaching hospitals in a developing country setting, we prefer to set a fairly simple structural model of a one-to-one relationship of the latent variables, i.e., operational flexibility, employee engagement, management capability, and hospital performance. The items were loaded onto respective factors of operational flexibility consisting of: input flexibility (was measured by 5 items), outcome flexibility (was measured by 5 items), process flexibility (was measured by 3 items), employee engagement (was measured by 12 items), management capability (was measured by 6 items), hospital performance consisting of process orientation (was measured by 9 items), work condition (was measured by 3 items), clinical quality (was measured by 4 items), patient satisfaction (was measured by 4 items), financial performance (was measured by 3 items), and operational efficiency (was measured by 4 items). The reliability of each is influenced by random measurement error; as indicated by the associated error term, each of these observed variables is regressed on its respective factor.

Hair Jr. [30] explained the three important types of modeling strategies that were emphasized, namely the model development strategy, the confirmatory modeling strategy, and the competing modeling strategy. Each of these three models represents a slightly different approach to modeling. The confirmatory approach is the most straightforward strategy. As understood by the name, the confirmatory factor approach allows researchers to define and unify a single model consisting of a specified number of hypothesis relationships and built according to real theories, and then apply SEM to assess the adequacy and accuracy of the model. In other words, the process is the confirmatory factor to obtain adequate support that the structural theoretical model fits and largely represents data. Secondly, the model strategy revolves to achieve testing of several examined models, i.e., comparing alternative models with aggregate models. Evaluating all the resulting models will lead to the best model that can represent the study data collected and is considered to be much stronger than testing only one model. The last stage is the work to develop a model strategy that basically adopts the basic theoretical framework and follows the adequacy and reasonableness of improving the framework through some adjustments to structural or measurement models. The model begins with a building based on theoretical provisions that is tested empirically and statistically using SEM modeling. Hence, it is possible to work on modifying the model based on the researcher’s judgments and opinions or according to suggestions based on the results of the modeling program used. Here, these re-specifications must be workable and theoretically applied in this study, and this work will follow the model development strategy.

The researchers performed structural equation modeling analyses using the AMOS 22 platform to calculate confirmatory factor analysis, and direct and indirect effects. Furthermore, SPSS 22 was used to compute descriptive statistics and a correlation matrix.

There are three common fit indices, namely Parsimonious Fit, Incremental Fit, and Absolute Fit have respective indices and acceptable standard values as given in Table 2.

**Table 2:** Categories of model fit

Category name	Name of index	Level of acceptance
Absolute Fit Index	RMSEA	RMSEA < 0.08
	GFI	GFI > 0.90
Incremental Fit Index	AGFI	AGFI > 0.90
	CFI	CFI > 0.90
	TLI	TLI > 0.90
	NFI	NFI > 0.90
Parsimonious Fit Index	Chi sq./df	Chi-Square/ df < 3.0

Source: [32]

## Results

### Assessing validity and reliability

Table 3 shows descriptive statistical measures such as mean, standard deviation, and Cronbach’s Alpha scores of the constructs. All of the construct factors yielded an alpha coefficient that exceeded the suggested acceptable 0.70 value [30]. From all the results presented for the value of Cronbach’s alpha coefficient, these results show that the questionnaire adopted in this study is well accepted (very reliable). In order to validate the instrument with the highest degree of verification, this study also utilized the analysis of moment structures software (AMOS) with maximum likelihood (ML) for data analysis (confirmatory factor analysis).

**Table 3:** Measurement of the variables of the hypothesized model

Construct	Dimension	Number of items	Mean	SD	Cronbach’s Alpha
Operational Flexibility	Input Flexibility	5	4.428	1.6074	0.922
	Process Flexibility	3	4.742	1.593	0.860
	Outcome Flexibility	5	4.52	1.588	0.922
	Total Operational flexibility items	13	4.535	1.583	0.945
Employee Engagement		12	3.378	1.052	0.874
Management Capability		6	4.4	1.464	0.929
Hospital Performance	Process orientation	9	3.35	0.996	0.890
	Workforce conditions	3	3.2	1.041	0.681
	Clinical quality	4	3.297	0.973	0.776
	Patient satisfaction	4	3.292	1.034	0.809
	Operational efficiency	4	3.159	1.0387	0.805
	Financial performance	3	3.174	1.0396	0.749
	Total Hospital performance items	27	3.27	0.981	0.934

### Inter-item correlations matrix

Table 4 presents the inter-item correlations among the study dimensions of the operational flexibility construct: employee engagement, management capability, and hospital performance dimensions. The correlations among the bivariate items show that there has been no item that is greater than 0.9. The results show that the correlations are at low levels, indicating that multicollinearity is not an issue in this study [33]. It gives initial evidence that the items are distinct from each other and represent the specified construct by using Pearson correlation coefficients. Most of the items are significant at the 0.01 level.

**Table 4:** Correlation matrix for all dimensions in the study

Correlations											
Study variables	1	2	3	4	5	6	7	8	9	10	11
1- Management Capability	1										
2- Employee Engagement	.562^a^	1									
3- Input Flexibility	.344^a^	.439^a^	1								
4- Outcome Flexibility	.285^a^	.388^a^	.624^a^	1							
5- Process Flexibility	.223^a^	.416^a^	.709^a^	.707^a^	1						
6- Process Orientation	.420^a^	.515^a^	.464^a^	.515^a^	.466^a^	1					
7- Work Condition	.380^a^	.384^a^	.335^a^	.298^a^	.267^a^	.492^a^	1				
8- Clinical Quality	.395^a^	.426^a^	.381^a^	.324^a^	.320^a^	.549^a^	.552^a^	1			
9- Patient Satisfaction	.386^a^	.487^a^	.366^a^	.420^a^	.359^a^	.576^a^	.512^a^	.618^a^	1		
10- Operational efficiency	.338^a^	.473^a^	.366^a^	.426^a^	.396^a^	.546^a^	.489^a^	.505^a^	.642^a^	1	
11- Financial Performance	.289^a^	.344^a^	.357^a^	.313^a^	.290^a^	.427^a^	.428^a^	.504^a^	.496^a^	.568^a^	1

^a^Correlation is significant at the 0.01 level (2-tailed)

The findings (Tables 3 and 4) demonstrated positive correlations between the studied operational flexibility dimensions, employee engagement, management capability, and hospital performance dimensions. The values of means and standard deviations were as follows: Input Flexibility (mean = 4.428, SD: 1.6074), Process Flexibility (mean = 4.742, SD: 1.593), Outcome Flexibility (mean = 4.52, SD: 1.588), Employee Engagement (mean = 3.378, SD: 1.052), Management Capability (mean = 4.4, SD: 1.464), Process Orientation (mean = 3.35, SD: 0.996), Workforce Conditions (mean = 3.2, SD: 1.041), Clinical Quality (mean = 3.297, SD: 0.973), Patient Satisfaction (mean = 3.292, SD: 1.034) Operational Efficiency (mean = 3.159, SD: 1.0387), and Financial Performance (mean = 3.174, SD: 1.0396). Significant strong correlations were found between operational flexibility dimensions’ practices, employee engagement, management capability, and hospital performance dimensions’ practices. Examples of these correlations were: Input Flexibility and Employee Engagement (*r* = 0.439**, *p* = 0.000), Process Flexibility and Employee Engagement (*r* = 0.416, *p* = 0.000), Management Capability and Process Orientation (*r* = 0.420, *p* = 0.000), and Employee Engagement and Process Orientation (*r* = 0.515, *p* = 0.000). All of the results shown in Table 4 provided strong support for the relationships examined in this study.

### Reliability, validity and confirmatory factor analysis

To verify the validity and reliability of the study instruments, both the composite reliability (CR) and average variance extracted (AVE) were used. The CRs were over the threshold of 0.70, and the AVEs of all measures are greater than 0.5, as reported in Table 5. This shows the convergent validity of the constructs. Moreover, the loading factor for all items was above 0.60 or a *p*-value less than 0.05.

**Table 5:** Average Variance Extracted (AVEs) and Composite Reliability (CRs)

	Composite Reliability (CRs)	Average Variance Extracted (AVEs)
Operational Flexibility	0.968	0.70
Management Capability	0.899	0.683
Employee Engagement	0.925	0.503
Hospital Performance	0.968	0.530

Therefore, all the items used were significant in measuring the study variables, following the rule of Hair et al. [30]. A confirmatory factor analysis was conducted to assess the model. The findings showed that the data fit the model well: χ^2^/df = 2.157; comparative fit index (CFI) = 0.944; Tucker–Lewis Index (TLI) = 0.938; and root mean square error of approximation (RMSEA) = 0.049; and GFI = 0.876; IFI = 0.944; NFI = 0. 901.

### Hypotheses testing

Table 6 presents the direct and indirect impact between the study variables.

**Table 6:** Direct and indirect impact for the structural model

**Direct impact for the Second order**						
**Dependent variable**		**Independent variable**	**Standardized coefficient**	**Lower Bound**	**Upper Bound**	***P*** **Values**
Management Capability	<−--	Operational Flexibility	.335	0.066	0.124	***
Employee Engagement	<−--	Operational Flexibility	.324	0.044	0.073	***
Employee Engagement	<−--	Management Capability	.527	0.288	0.302	***
Hospital Performance	<−--	Operational Flexibility	.372	0.115	0.212	***
Input (Operational Flexibility)	<−--	Operational Flexibility	.823	0.266	0.319	***
Outcome (Operational Flexibility)	<−--	Operational Flexibility	.840	0. 245	0.432	***
Hospital Performance	<−--	Employee Engagement	.367	0.575	0412	***
Hospital Performance	<−--	Management Capability	.148	0.087	1.268	.006
Process Flexibility	<−--	Operational Flexibility	.879	1.000	1.000	F
Process Orientation	<−--	Hospital Performance	.743	2.655	3.391	***
Work condition	<−--	Hospital Performance	.664	1.000	1.000	F
Clinical Quality	<−--	Hospital Performance	.741	1.245	1.553	***
Financial Performance	<−--	Hospital Performance	.643	0.888	1.158	***
Patient Satisfaction	<−--	Hospital Performance	.802	1.471	1.830	***
**Indirect impact for the Structural Model**						
**Dependent variable**	**Mediating variable**	**Independent variable**	**Standardized coefficient**	**Lower Bound**	**Upper Bound**	***P*** **Values**
Hospital Performance	Employee engagement	Operational Flexibility	0.102	0.075	0.140	0.002
Hospital Performance	Management capability	Operational Flexibility	0.031	0.023	0.44	0.001
Hospital performance	Employee engagement	Management Capability	0.311	0.179	0.454	0.003

Model fitting results: χ^2^/df = 2.157; comparative fit index (CFI) = 0.944; Tucker–Lewis Index (TLI) = 0.938; and root mean square error of approximation (RMSEA) = 0.049; and GFI = 0.876; IFI = 0.944; NFI = 0. 901

**Fig. 2:**
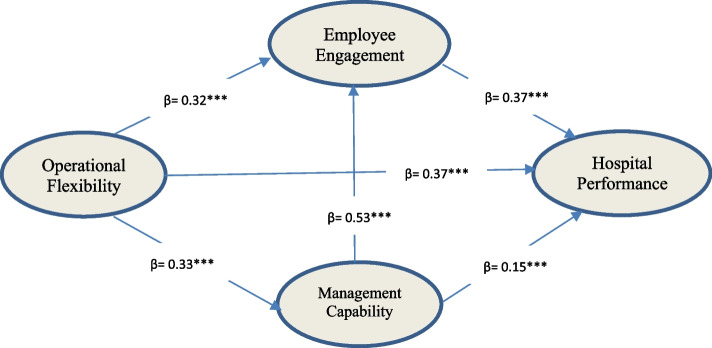
Structural equation modeling - standardized effects

To test our hypotheses, a structural equation model, as shown in Fig. 2, was built, including operational flexibility, management capability, employee engagement, hospital performance, and control variables. The fit indices of the SEM demonstrated a good fit of the model (Chi Square = 1164.540, DF = 540, TLI = 0.938, CFI = 0.944, IFI = 0.944, NFI = 0.901, RMSEA = 0.049 and *p*-value< 0.001). The results showed that operational flexibility had a direct and positive impact on hospital performance (β = 0.372; *p* < 0.001), and the relationship between operational flexibility and employee engagement was direct with a positive impact (β = 0.324; *p* < 0.001), the relationship between operational flexibility and management capability was positive with a clear direct impact (β = 0.335; *p* < 0.001), management capability had a significant result on the hospital performance (β = 0.148; *p* = 0.006), also management capability had a positive impact on employee engagement (β = 0..527; *p* = 0.006), and there is a positive relationship and direct impact between employee engagement and hospital performance (β = 0.367; *p* = 0.000). The above results lend support for H1 to H6.

On the mediating effects of employee engagement and management capability, the findings report that the indirect effect of operational flexibility on hospital performance through employee engagement (β = 0.102, *p* = 0.002) is significant, and the indirect effect of operational flexibility through management capability (β = 0.031, *p* = 0.001) is also significant but with no effect. Similarly, it was found that the indirect effect of management capability on hospital performance through employee engagement (β = 0.179, *p* = 0.003) was significant.

These results supported our hypotheses (H7, H8, and H9), which relate to the role of employee engagement as a mediation variable between operational flexibility and hospital performance, also as a mediation role between management capability and hospital performance. However, there was no clear indirect impact of operational flexibility on hospital performance through management capability.

## Discussion

This research study presented a novel approach that examined the impact of operational flexibility on hospital performance. Our results demonstrated that operational flexibility had a direct positive impact on hospital performance, employee engagement, and management capabilities. These findings are in alignment with what has been reported previously [1, 3, 14]. Alolayyan, Ali [14] found a strong association between operational flexibility and hospital performance, *r* = .836** with *p* < 0.05, and there was a significant impact of hospital operational flexibility on organization performance. Since operational flexibility has a significant effect on hospital performance, it is vital to determine the aggregate level of flexibility in hospitals against the individual level of each dimension of flexibility -input, process, and output-. This could be very useful in identifying where operational problems or performance opportunities exist. However, all three dimensions of flexibility are important in improving overall operational flexibility and organizational performance [3].

The results of the study showed that management capability had significant effects on hospital performance and had a direct positive impact on employee engagement. Also, there were positive and direct associations between employee engagement and hospital performance. Previous studies conducted in different industries showed that management capability is an important predictor of both financial and non-financial performance [24, 25]. When employees perceive that their management is supportive and encouraging, higher levels of engagement result. It has been found that employee engagement was positively associated with organizational citizenship behavior and performance, and there was a negative relationship between employee engagement and deviant [27]. Higher levels of staff engagement are strongly linked with higher levels of creativity and innovation because engaged employees usually perform their job tasks with passion [27, 34].

Furthermore, this study intended to examine the moderating effect of employee management and management capability on the relationship between operational flexibility and hospital performance, as well as the moderating effect of management capability on the relationship between operational flexibility and hospital performance. A key finding was the indirect effect of operational flexibility on hospital performance through employee engagement and the indirect effect of operational flexibility on hospital performance through management capability. Flexible organizations, through management competencies and employee engagement, motivate and develop employees to innovate and meet the highest and most sustained levels of organizational performance. Management with such capabilities is more able to acquire, generate, integrate, and reconfigure their valuable resources and competencies [3, 23, 35]. It has been argued that productivity and effectiveness in service organizations, including hospitals, are determined by staff engagement and involvement [36–38].

Growing technology and its extensive use in business operations have forced management to look towards highly motivated and engaged employees so that operational efficiency can be achieved. In the healthcare sector, employee engagement and the desire to achieve excellence are associated with organizational values related to psychosocial trust and safety, respect, fairness, and empowering leadership behavior [39]. Engaged employees who are emotionally and intellectually linked with the vision and values of the organization endeavor to be more productive. This is in correspondence with our model results in that employee engagement played a partial mediation effect between operational flexibility and hospital performance and played a partial mediation role also between management capability and hospital performance.

### Practice implications

The current study offers valuable insights for healthcare managers and leaders to understand the association between operational flexibility, management capability, and employee engagement and how these organizational components impact the enhancement of hospital performance. Our model makes an original contribution to operational flexibility in healthcare by understanding the aggregate level based on the three constructs (input flexibility, process flexibility, and outcome flexibility) of the transformational framework of flexibility [4]. Such activity contributes to the development of theories of flexibility management that can be used to better understand this complex concept. To date, this comprehensive understanding has not yet been empirically investigated [3], especially in healthcare organizations. Meanwhile, our validated model can be considered as a platform for further studies of operational flexibility in healthcare organizations, particularly in hospital settings.

Staff engagement as a key factor in operational flexibility and management capability directed the attention of hospitals’ management toward highly engaged and motivated staff so that better performance could be achieved. Thus, to improve or maintain a hospital’s performance, healthcare managers need to devote their efforts toward fostering staff engagement. Staff engagement and involvement are distinctively different from staff satisfaction and motivation. This distinction is critical in compliance with patient safety practices. Our findings highlight the need to invest more in management skills and competencies, as these have a bearing on staff engagement and hospital performance.

Operational flexibility and management capability are reflected in organizational agility. Nowadays, in extremely turbulent healthcare environments with a high level of complexity, uncertainty, and dynamism, organizational agility appears as a strategic solution to respond effectively and swiftly to these organizational challenges [40, 41]. For instance, although Jordan is one of the countries with limited financial capabilities, it has competent leadership and human resources and a high degree of harmony, which the whole world witnessed in the way Jordan dealt with the plight of Corona’s disease and how the spirit of sincerity and commitment was evident from all hospital crews. The medical staff’s engagement with management capability results in a high level of satisfaction with the operational flexibility of the health system. Altogether, this reflected how Jordanian hospitals have effectively and swiftly responded to the rapid epidemiological changes, which was reflected in more discretionary effort and distinguished hospitals’ performances.

The overarching conclusion of this empirical research is that significant progress has been achieved in hospital management, especially in terms of operational flexibility, management capability, and staff engagement. The results outline the performance outcomes of hospitals’ management in terms of clinical and nonclinical (financial) measures. Evidence-based management combined with experience can lead to successful strategies in healthcare management.

Finally, the choice of specialty hospitals as the survey setting creates a limitation in this study because it might hinder the generalizability of the findings. Testing our proposed model in different sectors (i.e., private) could be the subject of future research initiatives. Our model can also be enhanced in future empirical research. In addition to utilizing employee engagement and management capability as mediators, a number of other moderators could be used to investigate hospitals’ performance. For example, organizational culture and leadership style could be used to examine the relationship between operational flexibility, management capability, and hospital performance.

## Supplementary Information

**Additional file 1.** The study Questionnaire

## Data Availability

The datasets used and/or analysed during the current study available from the corresponding author on reasonable request.
