# Supplementary material for: Operational flexibility impact on hospital performance through the roles of employee engagement and management capability
Source: BMC Health Serv Res. 2023 Jan 9;23:19. doi: 10.1186/s12913-023-09029-y (PMC9827007; doi:10.1186/s12913-023-09029-y)
Supplement: Supplementary file 1 — Additional file 1. The study Questionnaire [file 12913_2023_9029_MOESM1_ESM.docx]

Additional file 1- The study Questionnaire

***Questionnaire to* *study and analyze (The Impact of Operational Flexibility on Hospital Performance through Employee Engagement and Management Capability)***

We are research group at Jordan University and Science Technology investigation the *Impact of Operational Flexibility on Hospital Performance through Employee Engagement and Management Capability* We would greatly appreciate your involvement in this project, through your completion of the attached survey. All information obtained from this questionnaire is confidential. It will be treated in strict confidence.

This survey seeks information about the *Operational Flexibility, Hospital Performance ,Employee Engagement and Management Capability* in your hospital.

In answering each question, please be as objective as possible remembering that biases sometimes “cloud” the real answer. You should not tick a box for example because the answer sounds more like what your “want” to hear but rather, indicate an answer which in your opinion accurately depicts the present situation in the hospital.

Your responses should reflect the overall situation in your hospital, not just in your particular area of responsibility. The validity of this research largely depends on the accuracy of your answers.

Thank you in advance for your participation in the completion of this questionnaire.

*Thank you very much!!!*

Best regards

The Research Team:

Jordan University of Science and Technology

Facility of Medicine

Health Management and Policy Department

Email: [mnalolayyan@just.edu.jo](mailto:mnalolayyan@just.edu.jo)

***Main Questionnaire***

Part A: Demographic information of employee

Profession

Medical managers and leaders

Supervisors

Senior administrative staff

Supervisors

Head of medical unit

Specialist and resident doctor

Nurse supervisor

Gender

Male:

Female:

Age

20-35 yrs.

36-50 yrs.

51-65 yrs.

66- 80 yrs.

Education

Diploma

Graduate degree

Postgraduate

Higher Specialty in Medicine

Name of hospital

A) Princess Basma Hospital,

B) King Abdullah University Hospital,

C) Princess Badea'a Hospital,

D) Princess Rahma Hospital,

B) Gynecology and Pediatrics’ Hospital-Almafraq

Number of years you have worked in this hospital

Less than 2 yrs.

2 - 5 yrs.

5 -10 yrs.

10 - 25 yrs.

*Read each statement and then use the seven -point response scale to indicate the extent to which you agree or disagree with each statement at the present time. Try to avoid leaving any item blank. If you don’t have an opinion on a statement or you don’t understand what it means, tick on number 4 (“Neutral”).*

**Rating scale:**

1- **Completely disagree** 2- **Moderately disagree**, 3- **Slightly disagree**, 4- **Neutral**  5- **Slightly agree,** 6- **Moderately agree**, 7- **Completely agree**

**Part C: The deployment of Operational Flexibility**

| **Input Flexibility** | **1** | **2** | **3** | **4** | **5** | **6** | **7** |
| --- | --- | --- | --- | --- | --- | --- | --- |
| Suppliers’ ability to respond to our request for changes in order mix. |  |  |  |  |  |  |  |
| Suppliers’ ability to respond to our request for changes in volume. |  |  |  |  |  |  |  |
| Suppliers’ ability to respond to our request for changes in delivery time. |  |  |  |  |  |  |  |
| Suppliers’ ability to respond to our request for changes in new services. |  |  |  |  |  |  |  |
| Suppliers’ ability to respond to our request for changes in service modifications. |  |  |  |  |  |  |  |
| **Process Flexibility** | **1** | **2** | **3** | **4** | **5** | **6** | **7** |
| Ability of our employees to handle a range of tasks. |  |  |  |  |  |  |  |
| Ability of the technologies to handle a wide range of operations. |  |  |  |  |  |  |  |
| Ability of our processes to perform procedures on patients in varied sequences. |  |  |  |  |  |  |  |
| **Outcome Flexibility** | **1** | **2** | **3** | **4** | **5** | **6** | **7** |
| Ability to expand capacity through overtime and/or temporary hiring. |  |  |  |  |  |  |  |
| Ability to produce a wide range of service lines within the period used by the hospital minimum planning. |  |  |  |  |  |  |  |
| Ability to introduce new and/or modifying existing services within |  |  |  |  |  |  |  |
| Ability to shorten service times for procedures. |  |  |  |  |  |  |  |
| Ability to produce varying levels of output at a profit within the minimum planning period used by the hospital. |  |  |  |  |  |  |  |

**Part B: The deployment of Hospital Performance**

*Read each statement and then use the five -point response scale to indicate the extent to which you agree or disagree with each statement at the present time. Try to avoid leaving any item blank. If you don’t have an opinion on a statement or you don’t understand what it means, tick on number 3 (“Neither agree nor disagree”).*

**1. Strongly disagree**

**2. Disagree**

**3. Neither agree nor disagree**

**4. Agree**

**5. Strongly agree**

| - **Process orientation** | | | | **1** | **2** | | **3** | **4** | **5** | |  |
| --- | --- | --- | --- | --- | --- | --- | --- | --- | --- | --- | --- |
| The work in our hospital is process oriented. | | | |  |  | |  |  |  | |  |
| Processes are documented and/or modeled | | | |  |  | |  |  |  | |  |
| Processes have defined owners (e.g. Case managers). | | | |  |  | |  |  |  | |  |
| Process owners (e.g. Case managers) are authorized to issue directives. | | | |  |  | |  |  |  | |  |
| The performance of all processes is reviewed on a regular basis. | | | |  |  | |  |  |  | |  |
| The results of performance measurement are used to change processes. | | | |  |  | |  |  |  | |  |
| Unsatisfactory processes are adapted. | | | |  |  | |  |  |  | |  |
| We have a defined procedure in place for changing processes. | | | |  |  | |  |  |  | |  |
| Thinking in department-spanning processes is encouraged through regular training. | | | |  |  | |  |  |  | |  |
| - **Workforce conditions** | | | | **1** | **2** | | **3** | **4** | **5** | |  |
| Our hospital has low turnover rate of staffs. | | | |  |  | |  |  |  | |  |
| Our employee satisfaction is high. | | | |  |  | |  |  |  | |  |
| Our hospital is known for high competency of staffs (good reputation). | | | |  |  | |  |  |  | |  |
| - **Clinical quality** | | | | **1** | **2** | | **3** | **4** | **5** | |  |
| Our hospital has hardly any redundant activities (e.g. redundant examinations). | | | |  |  | |  |  |  | |  |
| We have a low complication rate in our processes. | | | |  |  | |  |  |  | |  |
| Processes in our hospital meet the hospital's defined quality standards. | | | |  |  | |  |  |  | |  |
| Our hospital has a low re-hospitalization rate. | | | |  |  | |  |  |  | |  |
| - **Patient satisfaction** | | | | **1** | **2** | | **3** | **4** | **5** | |  |
| Our patients feel sufficiently informed about their treatment process. | | | |  |  | |  |  |  | |  |
| Our patients feel adequately cared for and advised. | | | |  |  | |  |  |  | |  |
| Our patients appreciate smooth processes and short waiting times in our hospital | | | |  |  | |  |  |  | |  |
| Our hospital has a small number of patient complaints. | | | |  |  | |  |  |  | |  |
| - **Operational efficiency** | | | | **1** | **2** | | **3** | **4** | **5** | |  |
| The processes in our hospital are efficient. | | | |  |  | |  |  |  | |  |
| Our hospital has short waiting times. | | | |  |  | |  |  |  | |  |
| Our hospital has a short length of stay. | | | |  |  | |  |  |  | |  |
| We discharge our patients within the average length of stay. | | | |  |  | |  |  |  | |  |
| - **Financial performance** | | | | **1** | **2** | | **3** | **4** | **5** | |  |
| Our hospital is generating the desired growth in revenues. | | | |  |  | |  |  |  | |  |
| Our process costs are low. | | | |  |  | |  |  |  | |  |
| Our costs are competitive (market-oriented). | | | |  |  | |  |  |  | |  |
| **(1 = strongly disagree, 2 = disagree, 3 = neutral, 4 = agree, 5 = strongly agree)** | | | | | | | | | | | |
| **Part C Employee Engagement** | **1** | **2** | **3** | | | **4** | | **5** | |  | |
| I know what’s expected of me at work. |  |  |  | | |  | |  | |  |  |
| I have the materials and equipment I need to do my work right. |  |  |  | | |  | |  | |  |  |
| At work I have the opportunity to do what I do best every day. |  |  |  | | |  | |  | |  |  |
| In the last seven days I have received recognition or praise for doing good work. |  |  |  | | |  | |  | |  |  |
| My supervisor, or someone at work seems to care about me as a person. |  |  |  | | |  | |  | |  |  |
| There is someone at work who encourages my development. |  |  |  | | |  | |  | |  |  |
| At work, my opinions seem to count. |  |  |  | | |  | |  | |  | |
| My associates or fellow employees are committed to doing quality work. |  |  |  | | |  | |  | |  |  |
| In the last six months, someone at work has talked to me about my progress. |  |  |  | | |  | |  | |  |  |
| Does the mission/purpose of your company make you feel your job is important? |  |  |  | | |  | |  | |  |  |
| In the last year, have you had opportunities to learn and grow? |  |  |  | | |  | |  | |  |  |
| Do you have a best friend at work? |  |  |  | | |  | |  | |  |  |

**1= Disagree Strongly; 2= Disagree Moderately; 3= Disagree Slightly; 4=Neutral; 5=Agree Slightly, 6= Agree Moderately; 7=Agree Strongly**

| **Part D - Management Capability** | **1** | **2** | **3** | **4** | **5** | **6** | **7** |
| --- | --- | --- | --- | --- | --- | --- | --- |
| We have integrated logistics systems. |  |  |  |  |  |  |  |
| We have cost control capabilities. |  |  |  |  |  |  |  |
| We have financial management skills. |  |  |  |  |  |  |  |
| We have human resource management capabilities. |  |  |  |  |  |  |  |
| We have accuracy of profitability and revenue forecasting. |  |  |  |  |  |  |  |
| We have marketing planning process. |  |  |  |  |  |  |  |
| We have integrated logistics systems. |  |  |  |  |  |  |  |
| We have cost control capabilities. |  |  |  |  |  |  |  |

***Thank you for your participation in this most important activity***

***Please return this questionnaire to the Researcher***
